# Positioning the first short axis slice for ventricular volume analysis

**DOI:** 10.1186/1532-429X-17-S1-T4

**Published:** 2015-02-03

**Authors:** Chris B Lawton, Nathan E Manghat, Mark Hamilton, Chiara Bucciarelli-Ducci

**Affiliations:** 1 CARDIAC MRI UNIT, Brstol Heart Institute, Bristol, UK

## Background

Ventricular volumetrics play a fundamental role in cardiac magnetic resonance imaging analysis. Accuracy is of course paramount, and thus crucial that the image data set is precise.This poster defines the correct positioning of the short axis (SA) cinematographic images, and the rationale behind it. Since most volume studies concentrate on the left ventricle (LV) slice positioning focuses on the mitral valve annulus.

## Methods

### Defining the ventricle

It is firstly important to define what you are measuring. The ventricles include the volume between the inlet/atrioventricular valve, the ventricular apex and outlet/ventriculo-arterial valve; imaging must cover this whole volume.

At analysis a debate may then arise about how much an inlet valve indents the ventricle, or an outlet valve indents the artery.

Typically the short axis slice has a thickness of 8-10 mm. It is recommended that the whole thickness of the slice be in the ventricle, otherwise partial voluming of the atrium will occur. Failure to do this may produce a 5-10% error in volumetrics.

### Positioning the first SA slice

**Figure 1:**
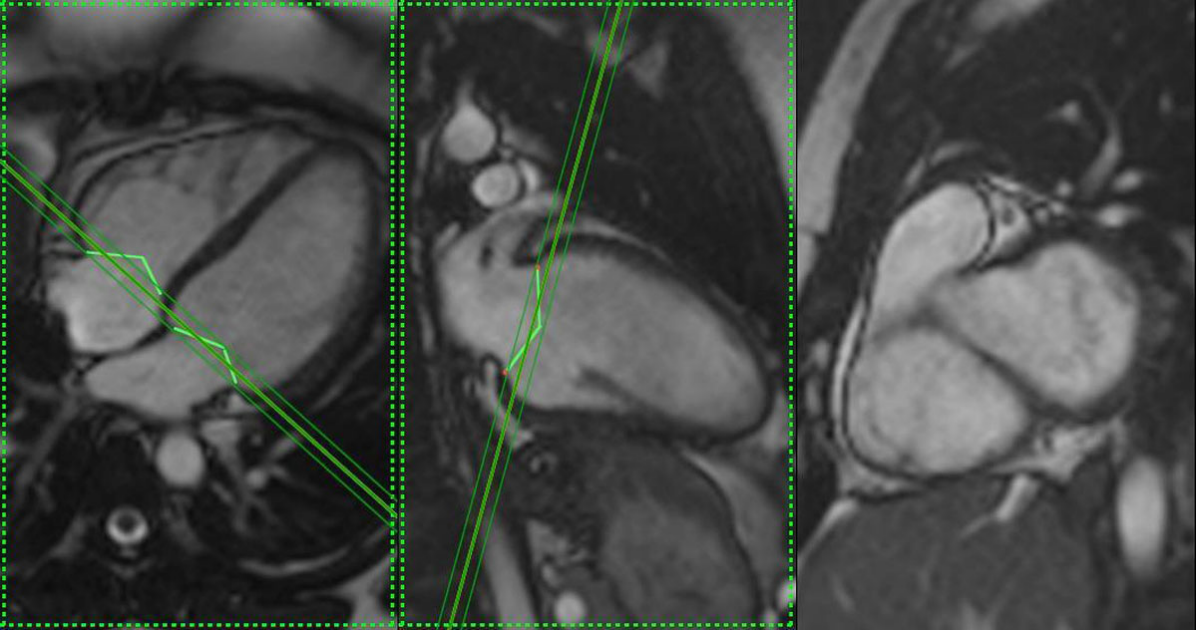
First SA slice is positioned with posterior aspect of the slice on the MV annulus.

Using the 4 chamber and 2 chamber long axis images, position the first short axis slice using the end diastolic time point 0. The posterior aspect of the slice thickness should be on the mitral valve annulus to include all ventricle on the first slice, but not atrium (Fig. 1) The slice is then advanced one step towards the apex and the next slice is imaged. This process continues until the apex is reached and the last image has no ventricular cavity.

### Right ventricle (RV) and SA planning

**Figure 2:**
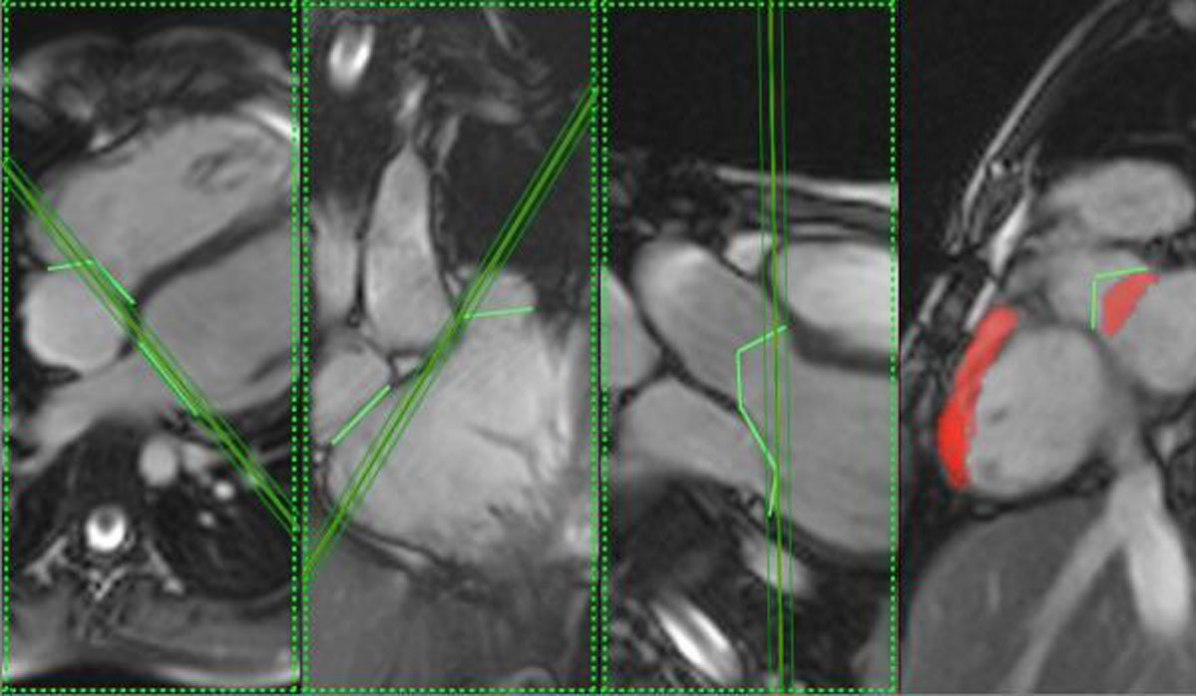
These images show the first SA slice and the omitted RV and LVOT if the slice is not moved toward the RA.

You will note in Fig. 2 the first SA slice (positioned as directed in Fig. 1) will be oblique to the tricuspid valve annulus and therefore a small volume of basal RV is frequently omitted in the cardiac volume. To avoid this, the slice can be moved one step posteriorly towards the right atrium to encompass all of the basal right ventricular volume. [This has an additional benefit of ensuring that no LV outlet (LVOT) is missed]. Check on a right 2 chamber, and right 3 chamber diastolic image for confirmation that this has occurred and also that the whole of the RV outlet has been included. Thus, slice 1 will be the most basal RV and slice 2 the most basal LV. This will heighten confidence when contouring the ventricles with respect to LV and RV basal and outlet demonstration.

## Results

None as a reference poster.

## Conclusions

Accurate definition of the structure to be measured and subsequent optimal positioning of the basal SA slice will lead to greater confidence that both ventricles are imaged in their entirety. This leads to more precise diagnosis and better study reproducibility.

## Funding

National Institute of Health Research. Biomedical Research Unit.

